# Oxytocin-induced increase in *N*,*N*-dimethylglycine and time course of changes in oxytocin efficacy for autism social core symptoms

**DOI:** 10.1186/s13229-021-00423-z

**Published:** 2021-02-23

**Authors:** Yasuhiko Kato, Hitoshi Kuwabara, Takashi Okada, Toshio Munesue, Seico Benner, Miho Kuroda, Masaki Kojima, Walid Yassin, Yosuke Eriguchi, Yosuke Kameno, Chihiro Murayama, Tomoko Nishimura, Kenji Tsuchiya, Kiyoto Kasai, Norio Ozaki, Hirotaka Kosaka, Hidenori Yamasue

**Affiliations:** 1 Department of Psychiatry, Hamamatsu University School of Medicine, 1-20-1 Handayama, Higashiku, Hamamatsu City, 431-3192 Japan; 2 Department of Psychiatry, Nagoya University Graduate School of Medicine, 65 Tsurumai-cho, Showa-ku, Nagoya, Aichi 466-8550 Japan; 3 Research Center for Child Mental Development, Kanazawa University, 13-1 Takara-machi, Kanazawa, 920-8640 Japan; 4 Department of Child Neuropsychiatry, School of Medicine, The University of Tokyo, 7-3-1 Hongo, Bunkyo-ku, Tokyo, 113-8655 Japan; 5 United Graduate School of Child Development, Osaka University, Kanazawa University, Hamamatsu University School of Medicine, Chiba University and University of Fukui, Osaka/Kanazawa/Hamamatsu/Chiba/Fukui, Japan; 6 Department of Neuropsychiatry, School of Medicine, The University of Tokyo, 7-3-1 Hongo, Bunkyo-ku, Tokyo, 113-8655 Japan; 7 Department of Neuropsychiatry, Faculty of Medical Sciences, University of Fukui, Eiheiji, Fukui 910-1193 Japan

**Keywords:** Asperger, Autism, Clinical trial, Developmental disorders, Facial expression, Metabolomics, Neuropeptide, *N*,*N*-Dimethylglycine, Oxytocin, Plasticity

## Abstract

**Background:**

Oxytocin is expected as a novel therapeutic agent for autism spectrum disorder (ASD) core symptoms. However, previous results on the efficacy of repeated administrations of oxytocin are controversial. Recently, we reported time-course changes in the efficacy of the neuropeptide underlying the controversial effects of repeated administration; however, the underlying mechanisms remained unknown.

**Methods:**

The current study explored metabolites representing the molecular mechanisms of oxytocin’s efficacy using high-throughput metabolomics analysis on plasma collected before and after 6-week repeated intranasal administration of oxytocin (48 IU/day) or placebo in adult males with ASD (*N* = 106) who participated in a multi-center, parallel-group, double-blind, placebo-controlled, randomized controlled trial.

**Results:**

Among the 35 metabolites measured, a significant increase in *N*,*N*-dimethylglycine was detected in the subjects administered oxytocin compared with those given placebo at a medium effect size (false discovery rate (FDR) corrected *P* = 0.043, *d* = 0.74, *N* = 83). Furthermore, subgroup analyses of the participants displaying a prominent time-course change in oxytocin efficacy revealed a significant effect of oxytocin on *N*,*N*-dimethylglycine levels with a large effect size (*P*_FDR_ = 0.004, *d* = 1.13, *N* = 60). The increase in *N*,*N*-dimethylglycine was significantly correlated with oxytocin-induced clinical changes, assessed as changes in quantifiable characteristics of autistic facial expression, including both of improvements between baseline and 2 weeks (*P*_FDR_ = 0.006, *r* = − 0.485, *N* = 43) and deteriorations between 2 and 4 weeks (*P*_FDR_ = 0.032, *r* = 0.415, *N* = 37).

**Limitations:**

The metabolites changes caused by oxytocin administration were quantified using peripheral blood and therefore may not directly reflect central nervous system changes.

**Conclusion:**

Our findings demonstrate an association of *N*,*N*-dimethylglycine upregulation with the time-course change in the efficacy of oxytocin on autistic social deficits. Furthermore, the current findings support the involvement of the *N*-methyl-D-aspartate receptor and neural plasticity to the time-course change in oxytocin’s efficacy.

*Trial registration*: A multi-center, parallel-group, placebo-controlled, double-blind, confirmatory trial of intranasal oxytocin in participants with autism spectrum disorders (the date registered: 30 October 2014; UMIN Clinical Trials Registry: https://upload.umin.ac.jp/cgi-open-bin/ctr_e/ctr_view.cgi?recptno=R000017703) (UMIN000015264).

## Background

Intranasal administration of oxytocin is a potential novel treatment for autism spectrum disorder (ASD) core symptoms, which currently have no established therapy [1, 2]. Although the beneficial effects of single-dose oxytocin on measures of ASD core symptoms have been consistently reported across studies [3–8], previous studies on the repeated administration of oxytocin have reported inconsistent findings, impeding further development of oxytocin as an approved medication [9]. Recently, we found a progressive deterioration in the efficacy of oxytocin [10, 11] and proposed that this phenomenon may account for the reported inconsistencies in the effect of repeated administration. Elucidating the mechanisms underlying the time-course change in the efficacy of repeated oxytocin administration may help advance the development of oxytocin-based therapy for ASD core symptoms.

Uncovering the interaction of oxytocin with other molecular systems is key to optimizing oxytocin-based therapies, including the identification of co-therapeutic agents [12]. We previously reported differential neurochemical effects of repeated oxytocin administration compared with acute treatment. Repeated administration specifically impacted the glutamatergic system, including the *N*-methyl-D-aspartate (NMDA) receptor [10, 13]: Repeated oxytocin administration, unlike acute oxytocin, significantly decreased the glutamatergic metabolite levels in the medial prefrontal cortex of participants with ASD. The decreases were inversely and specifically associated with oxytocin-induced improvements of medial prefrontal functional MRI activity during a social judgment task and not with changes during placebo administration. Furthermore, in wild-type mice, we found that repeated administrations of oxytocin reduced medial frontal transcript expression of NMDA receptor type 2B, unlike acute oxytocin. Previous animal studies also support the existence of interactions between oxytocin and glutamatergic neurotransmission [14, 15]. In addition, the time-course change in the efficacy of repeated oxytocin was detected with our unique dataset employing 2-week longitudinal assessments of objectively quantified measures of ASD social deficits [11]. Our facial expression analysis was based on videos recording during only a few minutes of activity in ADOS to quantify ASD-related social deficits [11, 16], whereas the entire ADOS requires 40–60 min to administer [17]. Although ADOS and gaze observation [18] are not optimized for longitudinal and repeated assessments in individuals with ASD, facial expression analysis is easily repeatable in longitudinal assessments [11, 16]. Previous studies have suggested that the efficacy of oxytocin deteriorates over time, possibly suggesting a potential underlying molecular mechanism [19, 20], such as a downregulation of oxytocin receptors [21, 22], or the glutamatergic system [10]. However, to the best of our knowledge, the relationship between the time-course change in efficacy and oxytocin-induced changes in molecular pathways has not yet been examined. In addition, potential links between oxytocin and other molecular systems, other than the glutamatergic system, have not been examined.

In the present study, we explored the interaction between oxytocin and molecular systems by analyzing oxytocin-induced changes using high-throughput metabolomics, which can quantify various metabolites related to the glutamatergic system as well as other molecular systems, such as the cholinergic and serotonergic systems. As the metabolomic panel, we selected a capillary electrophoresis system with an Agilent 6210 time-of-flight mass spectrometer (CE-TOFMS, Agilent Technologies, Santa Clara, CA, USA), in which the detection limits for most amino acids and anionic species were improved several-fold on average, and as much as 65-fold over previously reported values for the CE-quadrupole mass spectrometer [23]. Metabolite concentrations were quantified using plasma samples collected from participants before and after repeated administration of oxytocin or placebo in our previous multi-center, parallel-group, placebo-controlled, double-blind, confirmatory trial of intranasal oxytocin in adult males with high-functioning ASD [11, 24]. To the best of our knowledge, no previous study conducted metabolomic analyses before and after oxytocin administrations. Based on previous studies [10, 13], we hypothesized associations of the effects of oxytocin with changes in amino acids associated with glutamatergic transmission and also explored these relationships in other metabolites. Furthermore, by utilizing repeatable and quantifiable behavioral outcome measures, we explored the molecular mechanisms underlying the time-course changes in oxytocin efficacy on ASD.

## Methods

### Experimental design and participants

In the current study, we analyzed plasma samples collected from participants in our previous multi-center, parallel-group, placebo-controlled, double-blind, confirmatory trial of intranasal oxytocin in adult males with high-functioning ASD. The trial sites were the University of Tokyo Hospital, Nagoya University Hospital, Kanazawa University Hospital, and University of Fukui Hospital in Japan (UMIN000015264) [24]. The details of this trial are described elsewhere [11, 24]. Briefly, the inclusion criteria of this trial were as follows: (1) 18–54 years of age; (2) male; (3) diagnosis of autistic disorder, Asperger’s disorder, or pervasive developmental disorders not otherwise specified (PDD-NOS) based on DSM-IV-TR; (4) score exceeding the cut-off value (i.e., [10]) for qualitative abnormalities in social reciprocity on Autism Diagnostic Interview—Revised (ADIR) [25]; and (5) full IQ above 80 and verbal IQ above 85 based on WAIS—Third Edition (WAIS-III) [26]. The exclusion criteria were as follows: (1) primary psychiatric diagnosis other than ASD; (2) instable comorbid mental disorders (e.g., instable mood or anxiety disorder); (3) changes in medication or doses of psychotropics within 1 month before randomization; (4) current medication with more than two psychotropics; (5) current pharmacological treatment for comorbid attention-deficit/hyperactivity disorder; (6) history of repeated administrations of oxytocin; (7) history of hyper-sensitivity to oxytocin; (8) history of traumatic brain injury with loss of consciousness for longer than 5 min or seizures; or (9) history of alcohol-related disorders, substance abuse, or addiction. Open to the public recruitment and the processes testing eligibility are explained in detail elsewhere [24].

A total of 106 men with high-functioning ASD were recruited between January 2015 and March 2016. Among these participants, 94 were psychotropic-free other than oxytocin during the all trial period, while 12 continued their medications with psychotropic during the trial period (four antidepressants, four antipsychotics, two mood stabilizers, and two hypnotics). The diagnosis for subtypes of participants with ASD was autistic disorder (*N* = 83), Asperger’s disorder (*N* = 12), and PDD-NOS (*N* = 11).

### Intervention

The participants received administrations of oxytocin (48 IU/day) or placebo in the morning and afternoon during 6 weeks [24]. The placebo contained all of the inactive ingredients in order to control for any effect of substances other than oxytocin. On the last day of the 6-week administration period, data, including peripheral blood and clinical evaluations including Autism Diagnostic Observation Schedule (ADOS) [17], were collected from the participants. These endpoint clinical assessments were started 15 min after the last administration of intranasal oxytocin or placebo. All participants were sufficiently trained with identical instructions for intranasal administration, and the procedure of intranasal administration was evaluated at each 2-week assessment point. A self-report daily record was utilized to record treatment adherence.

### Randomization and masking

Drug administration was randomly assigned the participants to the oxytocin or placebo group in a one-to-one ratio by the manager of randomization and masking based on a computer-generated randomized order. The randomization was stratified based on the trial site and median score of ADIR (< 18 or ≥ 18, defined based on the results from our preliminary trial [27]). Spray bottles with the same visual appearance were utilized to store both active drug and placebo (Victoria Pharmacy, Zurich, Switzerland). The manager covered the labels of spray bottle to keep oxytocin or placebo blind to all the clinicians, assessors, their families, and participants. Registration, allocation, and data management procedures were defined separately [24].

### The main outcome of the current study

The main outcome of the current study was metabolite concentrations in plasma samples collected at baseline, immediately before the first administration of oxytocin or placebo, and at endpoint, 60 min after the last administration of oxytocin or placebo at 6 weeks from baseline. Peripheral blood samples were collected from the participants, while they were fasting (> 3 h without any meals or nutritious drinks) during the daytime. The blood sampling procedure was conducted by experienced physicians. Plasma was isolated with centrifugation at 1,600 g for 15 min at 4 °C and stored within 30 min after blood collection at − 80 °C until assay (see details in Additional file 1: “Standard operation paper for blood collection and processing in JOIN-Trial_in_English”). The plasma samples were collected from January 2015 to April 2016 and assayed with CE-TOFMS in January 2018. To the plasma samples (100 μL), 450 μL of methanol containing 10 mM each of methionine sulfone and 10-camphorsulfonic acid were added and mixed well. Then, 500 μL chloroform and 200 μL of Milli-Q deionized water (EMD Millipore, Billerica, MA, USA) were added. The solution was centrifuged at 2,300*g* for 5 min at 4 °C. Then, to remove proteins, a 400-μL aliquot of the supernatant was centrifugally filtered with a 5-kDa cut-off filter (Human Metabolome Technologies Inc., Tsuruoka, Japan). The filtrate was centrifugally concentrated in a vacuum evaporator and dissolved in 50 μL of Milli-Q water containing reference compounds before mass spectrometry analyses.

Plasma samples were measured using a capillary electrophoresis system with an Agilent 6210 time-of-flight mass spectrometer (CE-TOFMS, Agilent Technologies, Santa Clara, CA, USA) [28]. Customized proprietary software (MathDAMP) was utilized to process raw data files acquired from CE-TOFMS [29]. To identify target metabolites, their mass-to-charge ratio (m/z) values and migration times were matched with the annotation table of the metabolomics library (The Basic Scan metabolomics service of Human Metabolome Technologies Inc.) [30]. The relative area was defined by dividing all peak areas with the area of the internal standard. The definition of relative areas allowed avoidance of mass-spectrometry detector sensitivity bias and injection-volume bias across multiple measurements and normalization of the signal intensities. Based on the peak area of internal controls of each metabolite, the absolute quantities of 110 pre-determined major metabolites can be measured with analysis by CE-TOFMS in our system. We used the absolute quantities obtained with CE-TOFMS as metabolite concentrations in plasma samples.

### Other outcome measures of oxytocin efficacy

To examine their relationship to metabolite concentrations, we also included six additional outcomes found to be significant effects of oxytocin in this trial [11, 24] as well as in previous trials [11, 27]. The six clinical and behavioral indices of oxytocin efficacy were as follows: (i) ADOS repetitive behavior = changes in the ADOS repetitive score between baseline and 6-week endpoint of oxytocin administration (endpoint − baseline). ADOS is a standard diagnosis tool for ASD but recently has been increasingly adopted as a primary outcome in ASD-related trials [24, 27, 31–35]. (ii) Gaze fixation time on socially relevant regions = changes in the percentage of gaze fixation time on the eye region of a talking face presented on a video monitor, between baseline and 6-week endpoint (endpoint − baseline), which were measured with Gazefinder, a validated all-in-one eye-tracking system, for a few minutes subsequent to the ADOS sessions using the standardized and validated method described details in elsewhere [18, 24, 36, 37](JVC KENWOOD Corporation, Yokohama, Japan). (iii, iv, v, and vi) log-PDF_mode_ of quantified facial expression production of a neutral face during 0–6, 0–2, 2–4, and 4–6 weeks = changes in the natural logarithm of the mode of the probability density function of neutral facial expression intensity during a semistructured situation conducted during a few minutes of social interaction in the “*Cartoons*” activity in ADOS module 4. The data were quantified using a dedicated software program [38–40] (FaceReader version 6·1, Noldus Information Technology Inc., Wageningen, The Netherlands) using a validated method previously described in detail elsewhere [11, 16]. In addition to baseline and the 6-week endpoint, facial expression was assessed every 2 weeks as changes in log-PDF_mode_ of neutral facial expression between each assessment point (i.e., (iii) 6 weeks–baseline, (iv) 2 weeks–baseline, (v) 4 weeks–2 weeks, and (vi) 6 weeks–4 weeks). The log-PDF_mode_ for neutral facial expression is considered to reflect variation in facial expression [16] and can be characterized as a repeatable, objective, and quantitative measure of ASD-related social deficit.

### Classification of participants according to time-course change in the efficacy of oxytocin

To investigate the molecular mechanisms underlying the time-course change in the efficacy of repeated oxytocin administration, we defined a subgroup of the oxytocin-administered group comprising participants exhibiting a prominent time-course change. The rationale of this classification was based on our previous findings on the time course of oxytocin-induced quantitative changes in facial expression in ASD, which exhibited maximum efficacy at 2 weeks and deterioration of efficacy from 2 to 6 weeks [11]. Using this classification, we expected to detect metabolites related to the characteristics of participants with prominent time-course changes in the clinical effects of oxytocin. Individuals showing reduction of log-PDF_mode_ of neutral facial expression (i.e., improvement in ASD core symptom) from baseline to 2 weeks and increase of log-PDF_mode_ neutral facial expression (i.e., deterioration in ASD core symptom) from 2 to 6 weeks were classified as participants exhibiting a time-course change (Fig. 2c).

### Statistical analysis

Demographic and clinical information was compared using independent *t*-tests between placebo- and oxytocin-administered groups and between the placebo-administered group and the oxytocin-administered group exhibiting the time-course change.

We analyzed the effects of oxytocin on metabolite concentrations using independent *t*-tests for comparing changes from baseline to endpoint in metabolite concentrations during the 6-week administration period between the oxytocin-administered group and the placebo-administered group. Furthermore, because the change in metabolite levels over the 6-week oxytocin administration period could be associated with both clinical improvement and potential attenuation of oxytocin effectiveness, differences in changes in metabolite levels were also examined between the oxytocin-administered group displaying the time-course change in efficacy and the placebo-administered group. The independent *t*-tests were conducted for each metabolite, with absolute quantities successfully measured by CE-TOFMS measurement in at least 80% of all subjects (≧ 67 subjects) [41]. The Benjamini–Hochberg false discovery rate (FDR) correction for the number of metabolites tested was applied, and FDR-corrected *p* values of < 0.05 were considered statistically significant.

For the oxytocin-administered group, we calculated Pearson’s correlation coefficients for 6-week changes in outcomes versus changes in metabolite concentrations (identified as significant differences between the oxytocin and placebo-administered participants). The outcomes used in the correlation analysis were 6-week change in ADOS repetitive behavior, 6-week change in gaze fixation time on socially relevant regions, and log-PDF_mode_ of neutral facial expression change from baseline to 6 weeks. Furthermore, to clarify the relationships between the detected metabolite change and the time-course change in efficacy, changes in log-PDF_mode_ of neutral facial expression between each assessment point (i.e., 2 weeks–baseline, 4 weeks–2 weeks, and 6 weeks–4 weeks) were calculated and correlated with changes in metabolites using Pearson’s correlation coefficient. The Benjamini–Hochberg FDR correction for the number of outcomes tested was applied to adjust the results, and the statistical significance level was defined as FDR-corrected *p* values of < 0.05. STATA version 14.0 and GraphPad Prism 8.4.1 were employed to conduct all statistical analyses.

To assess whether the association between the efficacy of oxytocin and changes in the variability of quantified neutral facial expression between 0 and 2 weeks was mediated by changes in the level of DMG (a metabolite that exhibits a significant increase related to oxytocin administration), linear regression models were fitted according to the Baron and Kenny procedures for mediation analysis [42].

## Results

### Demographic information of participants

**Fig. 1:**
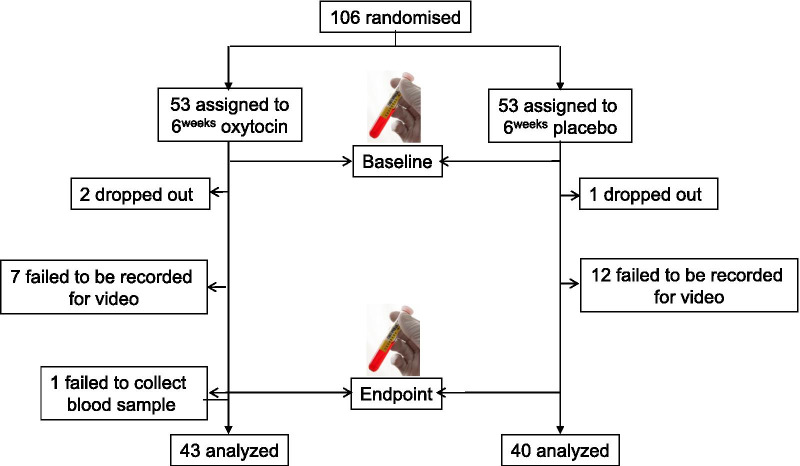
Participants flow in the current study

**Fig. 2:**
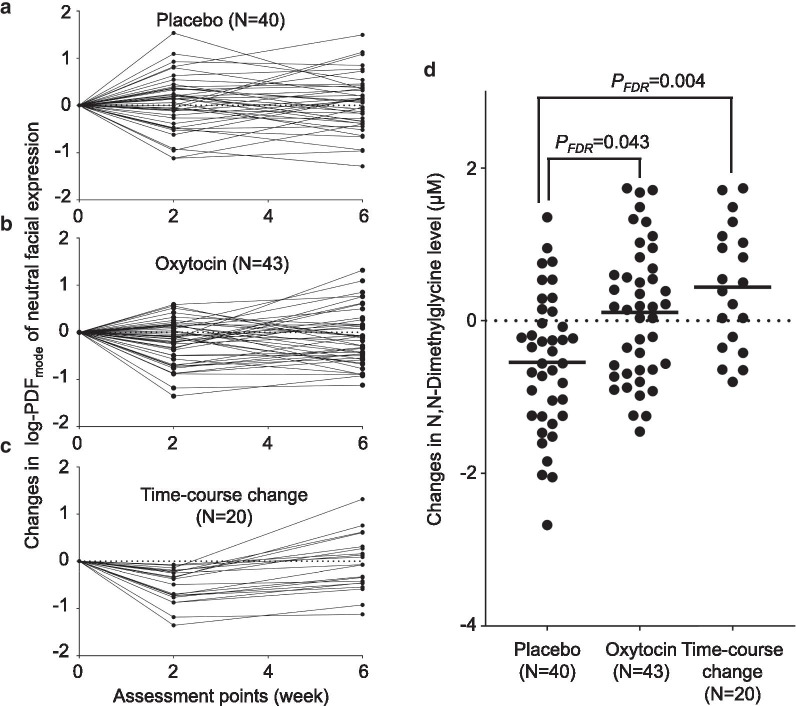
Effects of intranasal oxytocin on changes in metabolite concentrations and time-course change in effects of oxytocin. **a**–**c** Individual changes from baseline in the natural logarithm of the mode of the probability density function (log-PDFmode) of neutral facial expression intensity. Plots show changes from baseline of log-PDFmode of neutral facial expression intensity in participants administered placebo (**a**) or oxytocin (**b**). Among the participants administered with oxytocin, individuals showing reduction of log-PDFmode of neutral facial expression from baseline to 2 weeks and increase of log-PDFmode neutral facial expression from 2 to 6 weeks were classified as participants exhibiting a time-course change (**c**). **d** Plots show changes in plasma DMG levels during the 6-week administration of oxytocin or placebo. Bars indicate mean concentration change in each group

Detailed flow of participant is shown in Fig. 1. Two participants in the oxytocin group and one in the placebo group did not complete the trial because of withdrawal of consent or discontinuation of administration. Among the remaining 103 participants, after exclusion of subjects failing to be recorded in the ADOS [17] video recordings at any assessment point, 44 subjects in the oxytocin group and 40 subjects in the placebo group remained. One subject in the oxytocin group, not classified as exhibiting attenuation of oxytocin efficacy, was unable to provide a blood sample. In the end, a total of 83 individuals with ASD were analyzed to investigate relationships between the paradoxical attenuation of oxytocin efficacy and metabolite concentration changes (Fig. 1). Twenty of the 44 subjects in the oxytocin-administered group were classified into the time-course change group (Fig. 2). This classification of individuals with time-course attenuation was based on our previous findings on the time course of oxytocin-induced quantitative changes in facial expression in ASD which showed maximum efficacy at 2 weeks and deterioration of efficacy from 2 to 6 weeks [11] (Fig. 2c). No significant differences between the oxytocin- and placebo-administered participants or between the time-course change and the placebo groups were detected in background information, except for age between the time-course change group and the placebo group (*p* = 0.02) (Table 1).

**Table 1:** Demographic background and clinical characteristics of the participants

	Placebo-administered group (*N* = 40)		Oxytocin-administered group (*N* = 43)		Oxytocin versus placebo group	Time-course change group (*N* = 20)		Time-course change versus placebo group
	Mean	SD	Mean	SD	*p* value^c^	Mean	SD	*p* value^c^
Age (range)	26.4 (18–46)	6.9	28.2 (18–48)	7.4	0.26	30.9 (22–47)	6.6	0.02
Height, cm	172.2	5.7	170.5	6.4	0.21	170.5	6.1	0.30
Body weight, kg	66.7	11.5	67.3	11.5	0.80	69.5	11.2	0.37
SES^a^	2.8	1.2	3.2	1.0	0.08	3.3	1.1	0.12
Parental SES^a^	2.2	0.6	2.2	0.6	0.66	2.1	0.6	0.75
Handedness: Right/left	37/3		41/2			20/0		
IQ^b^								
Full IQ	110.3	14.1	105.2	13.7	0.10	106.4	14.6	0.31
Verbal IQ	116.5	14.3	110.7	13.2	0.06	110.8	13.4	0.14
Performance IQ	100.2	14.7	96.5	15.4	0.27	98.1	16.3	0.61
ADIR								
Social	21.9	5.2	21.2	5.0	0.57	21.7	4.4	0.91
Communication	16.3	3.4	15.6	3.9	0.36	15.8	3.6	0.56
Repetitive	5.9	2.3	5.3	2.6	0.36	5.3	2.6	0.41

SES, socioeconomic status; IQ, intelligence quotient; SD, standard deviation; ADIR, Autism Diagnostic Interview—Revised

^a^SES assessed using the Hollingshead scale. Higher scores indicate lower status

^b^Intelligence quotients were measured using the Wechsler Adult Intelligence Scale

^c^*p* values were calculated using the independent *t*-test

### CE-TOFMS measurement of metabolite concentrations

Using CE-TOFMS [28] analysis, which can measure absolute quantities of metabolite concentrations, among the 110 pre-selected metabolites, 50 were detected in the plasma samples. Of these 50 metabolites, three were not detected from the plasma samples collected at baseline or endpoint. Furthermore, 12 were excluded based on the rate of successful measurements (i.e., less than 80%, which was employed as the threshold in a previous study utilizing the same metabolomic panel [41], while 35 metabolites were measured in all (i.e., 100%) of the 166 plasma samples (Additional file 2: Figure 1). It has been reported that a large amount of missing data (greater than 10%) can bias the results of subsequent statistical analyses in medical research [43]. Thus, we used the concentrations of these 35 metabolites for further analyses.

### Metabolite concentration changes in participants with ASD

We examined the effects of oxytocin treatment on the levels of the 35 metabolites and found a significant increase in the levels of *N,N*-dimethylglycine (DMG) during the 6-week repeated administration of oxytocin compared with placebo after correction for multiple comparisons (*P*_FDR_ = 0.043, *d* = 0.74, *N* = 83) (Fig. 2d). Although the citric acids level was decreased during the 6-week administration of oxytocin compared with placebo (*P* = 0.029, *d* = 0.49, *N* = 83), the statistical significance did not survive correction (*P*_FDR_ = 0.51). No significant effects of oxytocin on changes in concentration of the remaining 33 metabolites were found (*P*_FDR_ > 0.57, Additional file 3: Table 1). Additional analyses confined to psychotropic-free subjects (*N* = 72) and subjects diagnosed with autistic disorder (*N* = 62) were conducted and confirmed that the statistical conclusions were not changed by considering these potential confounds with excluding subjects with any psychotropic medication (*N* = 11) or subjects diagnosed with Asperger’s disorder or pervasive developmental disorders not otherwise specified (PDD-NOS) (*N* = 21).

Next, to clarify whether the concentration change was related to clinical improvement or attenuation of efficacy, we examined the effects of oxytocin on metabolite levels in the subgroup of ASD individuals with time-course attenuation in efficacy. This subgroup analysis revealed a significant effect of oxytocin on DMG levels (*P*_FDR_ = 0.004, *d* = 1.13, *N* = 60) (Fig. 2d), but not on the levels of the remaining 34 metabolite levels (*P*_FDR_ > 0.80, Additional file 4: Table 2). Notably, the effect size of oxytocin on DMG levels was larger in the time-course change subgroup than in the oxytocin-administered group as a whole. Although the age of the time-course change group was significantly older than that of the placebo-administered group, the analyses, controlling age as covariate, did not impact the statistical conclusion (Additional file 5: Table 3). Additional analyses confined to psychotropic-free subjects (*N* = 54) and subjects diagnosed with autistic disorder (*N* = 45) also confirmed that the statistical conclusions were preserved.

**Fig. 3:**
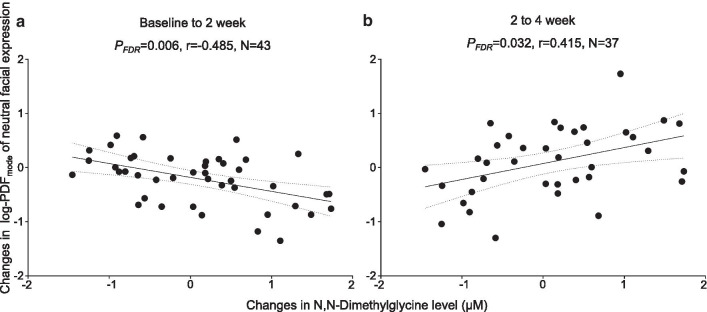
Relationship between oxytocin-related changes in metabolite concentration and time-course change in behavioral effect of oxytocin on autistic facial expression. Oxytocin-related increase in DMG level showed significant correlations with both the decrease in autistic facial expression, indexed as log-PDFmode of neutral facial expression, from baseline to 2 weeks (**a** *P*_FDR_ = 0.006, *r* =  − 0.485, *N* = 43) and the increase from 2 to 4 weeks (**b** *P*_FDR_ = 0.032, *r* = 0.415, *N* = 37) in participants with autism spectrum disorder. One participant in baseline to 2 weeks and seven participants in 2 to 4 weeks were excluded because of recording failure, defocused video images, or poor facial recognition rate at least one assessment point among these. Regression lines (solid) and 95% confidence band (dashed) were fitted using simple linear regression

We further conducted correlational analyses to clarify the relationship between the increased DMG levels and the clinical and behavioral effects of oxytocin. The analyses showed that the increase in DMG was significantly correlated with improvement indexed as change from baseline to 2 weeks in log-PDF_mode_ of neutral facial expression (*P*_FDR_ = 0.006, *r* =  − 0.485, *N* = 43) (Fig. 3a, Additional file 6: Table 4). Furthermore, the increase in DMG was also significantly related to change from 2 to 4 weeks in log-PDF_mode_ of neutral facial expression in the opposite direction (*P*_FDR_ = 0.032, *r* = 0.415, *N* = 37) (Fig. 3b). In contrast, no significant correlation between the increase in DMG and clinical or behavioral improvements, indexed as changes from baseline to 6 weeks in ADOS repetitive behavior, gaze fixation time on socially relevant regions, and log-PDF_mode_ of neutral facial expression (*P*_FDR_ > 0.65, Additional file 6: Table 4). In addition, no significant correlation was found between any clinical or behavioral change and change in DMG level in the placebo-administered group (*P*_FDR_ > 0.23). Additional correlational analyses confined to psychotropic-free subjects and subjects diagnosed with autistic disorder also confirmed that the statistical conclusions were preserved. The correlation between changes in oxytocin level and DMG level was additionally tested, revealing no significant correlation (*p* = 0.32).

The mediation analysis revealed that neither direct (*p* = 0.096) nor indirect effects (*p* = 0.235) were statistically significant, although the total effect of oxytocin on facial expression was significant (*p* = 0.026). Furthermore, by testing the mediating effect of DMG on the 6-week clinical effects of oxytocin, we confirmed that there was no significant indirect (i.e., mediating) effect of DMG (ADOS repetitive behavior: *p* = 0.609; gaze fixation time on socially relevant regions: *p* = 0.741) and that there were significant direct and total effects of oxytocin on these clinical measures (ADOS repetitive behavior: direct effect *p* = 0.017, total effect *p* = 0.015; gaze fixation time on socially relevant regions: direct effect *p* = 0.004, total effect *p* = 0.014).

## Discussion

The current parallel-group comparison of metabolites changes between the oxytocin- and placebo-administered groups revealed a significant increase in plasma DMG levels during the 6-week intranasal oxytocin treatment period. This change was prominent in the participants exhibiting a time-course change in oxytocin efficacy. Furthermore, the increase in DMG was associated with behavioral changes in autistic characteristics of quantified facial expression (i.e., improvements from baseline to 2 weeks and deteriorations from 2 to 4 weeks), although the increase in DMG was not related to improvements in clinical or behavioral outcomes during the 6-week administration period as a whole.

Here, we found a significant increase in DMG induced by oxytocin administration in the participants with ASD. DMG, a nutrient supplement and a partial agonist for NMDAR glycine binding sites, is the *N*,*N*-dimethylated derivative of glycine. DMG is a natural amino acid found in certain foods, such as beans, cereal grains, and liver. DMG has been marketed in vitamin B15 since 1975 and was subsequently isolated as a single nutritional supplement to serve as an athletic performance enhancer [44]. DMG is an important intermediary in the amino acid metabolism from choline and glycine betaine to sarcosine and glycine [45]. DMG can modulate NMDA receptors (NMDAR), because sarcosine (monomethylglycine) and glycine act as NMDAR co-agonists by occupation of glutamate binding sites in the NMDAR [46, 47]. A putative functional partial agonist for glycine sites of the NMDAR produces psychotropic effects [48]. A previous study reported that NMDAR is critical for development and rescuing ASD-like phenotypes observed in Shank2-mutant mice and that by modulating NMDAR, metabotropic glutamate receptor 5 may provide a novel treatment target for ASD [49]. DMG derivatives also exhibit pharmacological activities in the central nervous system, decreasing oxidative stress [50], improving immune responses [51], and exhibiting anticonvulsant activity in animal models [52]. Psychotropic effects of DMG have also been reported in animal studies as having antidepressant-like effects with reduction in ketamine-induced psychotomimetic behaviors [53] and exerting a preventing effect on NMDAR inhibitor-induced impairment in social recognition memory [54]. Several studies, including randomized controlled trials, have reported lower levels of plasma DMG [55] and clinical effects of administration of DMG [56–59] in individuals with ASD, although the results remain controversial. Our current study provides the first clinical evidence for a relationship between changes in DMG and oxytocin treatment in subjects with ASD. Together with previous animal studies showing interactions between central oxytocin and NMDAR such as central oxytocin release stimulated by NMDAR glycine site agonists [10, 60, 61], the current study supports the potential combination therapy of DMG or a NMDAR modulator and oxytocin for ASD.

DMG is a partial agonist at the glycine binding site of NMDA receptors. However, although DMG alone did not alter the NMDA receptor-mediated excitatory field potentials, DMG acts as an agonist at the glycine binding site of NMDA receptors in combination with glutamate [62]. Therefore, the agonist effect of DMG on NMDAR can be decreased with decreased medial prefrontal glutamate–glutamine concentration, and decreased NMDAR expression has been reported to occur during chronic administration of oxytocin [10]. As demonstrated in our previous study [11], the behavioral effects of oxytocin can be observed at a maximum of 2 weeks, with deterioration at 4 weeks, in a 6-week treatment period. Taken together, these findings suggest that in the acute phase (e.g., 0–2 weeks), increases in DMG induced with oxytocin administration can act as a partial agonist at the glycine binding site of NMDAR with glutamate [62]. In addition, administered oxytocin has clinical effects during the acute phase. In the chronic phase (e.g., 2–4 weeks), increases in DMG induced with administered oxytocin do not have an agonist effect on NMDAR under a decrement of glutamate [10]. As decreases in NMDAR induce decreased secretion of oxytocin [63], the clinical effects of oxytocin may also decrease. Although this interpretation is speculative, the current finding of increased DMG levels associated with oxytocin administration and its relationships with the emergence of positive behavioral effects of oxytocin between 0 and 2 weeks and the inverse deterioration of the effects of oxytocin on behavioral symptom, an autistic characteristic of facial expression, between 2 and 4 weeks is consistent with the interpretation. Furthermore, this notion is also consistent with the results of mediation analyses showing that both DMG increases and behavioral changes were associated with oxytocin administration in a parallel way, rather than clinical effects of oxytocin being mediated by increased DMG. Hence, our findings suggest that DMG and its interactions with NMDAR and glutamate are associated with modulation of oxytocin secretion and that the modulated secretion is associated with both emerging and deteriorating clinical effects of oxytocin, and further explain the lack of consistency in beneficial effects of chronic oxytocin in previous studies. Future studies will be needed to test this hypothesis in a design involving longitudinal assessment of DMG, glutamate, NMDAR, oxytocin levels, and behavioral evaluations.

The increase in DMG was most prominent in the ASD participants exhibiting a time-course change in oxytocin efficacy. In addition, although the increase in DMG was not related to clinical or behavioral improvements during the 6-week administration period as a whole, the increase was associated with improvements from baseline to 2 weeks and also with deterioration from 2 to 4 weeks, assessed as behavioral changes in quantified characteristics of autistic facial expression. The mediation analyses revealed that oxytocin affected DMG levels and quantified facial expression in a parallel way. Collectively, our findings indicate that both the upregulation of DMG and time-course changes in quantified social behavior are associated with the efficacy of oxytocin for ASD. Together with previous animal studies on the relationship between oxytocin efficacy and NMDAR-dependent neural plasticity [10, 64, 65], our present clinical study further supports an association between NMDAR and neural plasticity in time-course changes, such as improvement in and subsequent deterioration of oxytocin efficacy.

Previous animal studies support a relationship between oxytocin and neural plasticity via glutamatergic transmission—oxytocin enhances excitatory synaptic transmission [66] and facilitates long-term potentiation [64]. Our recent human clinical trial and animal study [10] further supports a relationship between NMDAR and oxytocin: Repeated administration of oxytocin downregulates medial prefrontal glutamatergic metabolites (i.e., *N*-acetylaspartate and glutamate–glutamine), measured with ^1^H-magnetic resonance spectroscopy, compared with acute oxytocin [13]. The decreases in these metabolite levels were negatively and specifically correlated with oxytocin-induced improvements in medial prefrontal function. Furthermore, we showed that repeated administration of oxytocin decreased expression of the transcript for NMDA receptor type 2B in the medial prefrontal region, in contrast to acute oxytocin, in wild-type mice [10]. The current study further shows a link between changes in NMDA and time-course change in the efficacy of repeated administration of oxytocin in individuals with ASD.

The present study with a peripheral metabolomics supports the possibility that changes in blood DMG level can briefly monitor the efficacy and its time course of oxytocin. Previous studies have suggested that metabolomics analyses are likely to sensitive for interactions among metabolite levels and the presence of a disorder such as ASD as well as factors such as severity of the disorder, comorbid conditions, diet, supplements, sex, genome, and other environmental factors [67]. Thus, metabolic signatures for psychiatric disorders could promote the identification of biomarkers for disease, for progression of disease or for response to therapy. In addition, it was proposed that metabolomics provides powerful tools for the process of drug discovery and development by providing detailed biochemical knowledge about drug candidates, their mechanism of action, therapeutic potential, and side effects [68, 69].

We found no significant correlations between increased DMG and changes in plasma oxytocin levels. A previous study reported that a substantial increase in oxytocin plasma levels 30 min after intranasal administration and group mean oxytocin plasma levels returned to baseline by 90 min post-administration [70]. In contrast, the time course of DMG levels after oxytocin administration is currently unknown. Because the time course of DMG changes is unlikely to exactly match that of oxytocin, the lack of correlation between the changes in DMG and oxytocin levels quantified with blood collected at 60 min after administration of oxytocin is not surprising.

### Limitations

There are several potential limitations to the current study. First, the participants in this study were all Japanese, adult, males with high-functioning ASD. Therefore, although the uniformity in demographic backgrounds enhanced the ability to detect metabolomics changes in the current study, the current findings should carefully be generalized to other clinical or non-clinical populations. Second, the metabolites changes caused by oxytocin administration were quantified using peripheral blood, and therefore may not reflect central nervous system changes. Further study is needed to clarify the interaction between oxytocin and molecular systems in the central nervous system. Third, considering the potential effects of nutrition on metabolite levels, we confirmed that there was no difference in BMI between the oxytocin and placebo groups. Furthermore, we tested correlations between BMI and changes in DMG levels and found no significant correlations. On the day of blood collection, all participants were fasting (> 3 h without consuming any meals or nutritious drinks) before collection from a peripheral vein, to reduce the effects of nutrition. However, it was reported that oxytocin can influence body weight, namely through reduction in food intake as well as increases in energy expenditure and/or lipolysis [71]. Because we did not measure body weight changes or quantify dietary content during the trial period, the possibility that changes in food intake during the 6-week administration period affected DMG levels cannot be completely ruled out. Future study is expected to collect information about individual’s diet and add it as a covariate in the analysis. Fourth, although blood samples collected from 2 and 4 weeks after the start of treatment need to be analyzed to further support the “time-course” relationship with *N*,*N*-DMG levels, metabolomic analysis on peripheral blood is difficult to repeat, mainly because of the high burden of repeated blood collection on clinical trial participants and the substantial financial cost of metabolomic analyses. Future study with repeated blood collections is needed to see whether there is a consistent/reliable increase in DMG levels following repeated administrations of oxytocin.

## Conclusions

In conclusion, the present high-throughput metabolomic analysis of plasma from a large-scale multi-center randomized controlled trial provides clinical evidence for an association between oxytocin-related increase in DMG and time-course changes in the efficacy of oxytocin for ASD social core symptoms. The results further support a contribution of NMDAR and neural plasticity to the time-course change. Our findings might suggest a potential optimization of oxytocin-based combinatorial therapy of an NMDAR modulator and oxytocin for ASD, such as for individuals showing deterioration in efficacy.

## Supplementary Information

**Additional file 1**. **Supporting Table 1**. Difference in changes of metabolites level between oxytocin and placebo.

**Additional file 2**. **Supporting Table 2**. Difference in changes of metabolites level between time-course change- and placebo-administered groups.

**Additional file 3**. **Supporting Table 3**. Difference in changes of metabolites level between time-course change- and placebo-administered groups with covariating age.

**Additional file 4**. **Supporting Table 4**. The relationship of the increased N,N-Dimethylglycine to clinical and behavioral effects of oxytocin.

**Additional file 5**. **Supplementary Figure 1**. The number of successful measurements using a capillary electrophoresis system with an Agilent 6210 time-of-flight mass spectrometer (CE-TOFMS).

**Additional file 6**. Standard operation paper for blood collection and processing in Japanese Independent Trial of Oxytocin.

## Data Availability

The data underlying the findings of this study are available from the corresponding author (H.Y.) on request from investigators providing a methodologically sound proposal and whose proposed use of the data has been approved by an independent review committee identified for this purpose. Maintenance of the identified dataset in the participants of clinical trials will be ended 5 years following article publication, but the deidentified data will be maintained indefinitely. The data are not publicly available due to them containing information that could compromise research participant privacy or consent.

## Abbreviations

ASD: Autism spectrum disorder
FDR: False discovery rate
NMDA: *N*-Methyl-D-aspartate
NMDAR: *N*-Methyl-D-aspartate receptors
PDD-NOS: Pervasive developmental disorders not otherwise specified
ADIR: Autism Diagnostic Interview—Revised
WAIS-III: WAIS—Third Edition
ADOS: Autism Diagnostic Observation Schedule
CE-TOFMS: Capillary electrophoresis system with an Agilent 6210 time-of-flight mass spectrometer
DMG: *N*,*N*-Dimethylglycine
