# Supplementary material for: Oxytocin-induced increase in N,N-dimethylglycine and time course of changes in oxytocin efficacy for autism social core symptoms
Source: Mol Autism. 2021 Feb 23;12:15. doi: 10.1186/s13229-021-00423-z (PMC7903697; doi:10.1186/s13229-021-00423-z)
Supplement: Supplementary file 1 — Additional file 1. Supporting Table 1. Difference in changes of metabolites level between oxytocin and placebo. [file 13229_2021_423_MOESM1_ESM.docx]

Standard operation paper for blood collection and processing in Japanese Independent Trial of Oxytocin

Created date: 2012/05/17

Update date: 2015/1/22

1. **Preparation before blood collection**

☐ Sample collection voucher

☐ Blood collection tube × 4 (☐ Blood count ×1; ☐Biochemistry ×1; ☐EDTA 7 ml × 2)

*Label*

For each subject

☐ Microtube × 6 (Oxytocin [4.5 ml] × 1 + Normal [2 ml] × 5)

☐ Re-plug cap for EDTA 7 ml blood collection tube × 3

☐ Set of blood collection tools × 1 (e.g., needle, syringe, tourniquet, alcohol cotton, adhesive plaster)

☐ Micropipette (1000 μl) × 1

☐ Cooling box (spread with ice) × 1

☐ Steel rack × 1


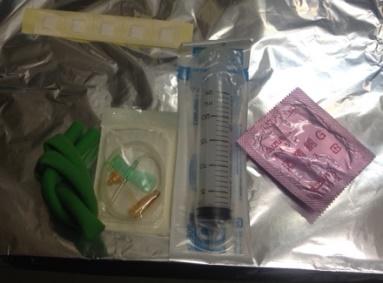

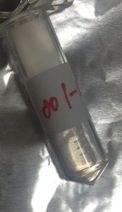
☐ Microtube stand × 1

2 ml microtube Set of blood collection tools

On the day, enter the laboratory approximately 30 minutes before blood collection for preparation.

1. ☐ Turn on the centrifuge and set the temperature to 4 °C and 1600 G (3000 rpm). (Please confirm the start of cooling by listening to the sound of the refrigerator motor. The refrigerator takes 15 to 20 minutes to cool down.)
2. Prepare for post-processing in the laboratory

・Disinfection with alcohol and RNAse inactivation for re-plug caps, micropipettes, steel racks, microtube stands, and desk surfaces. Place aluminum foil on the desk for dispensing tubes.

1. Prepare tools and wait

- Blood collection tools (e.g., needle, syringe, tourniquet, alcohol cotton, adhesive plaster)
- Blood collection tube × 5 / participant (blood count × 1; biochemistry × 1; EDTA 7 ml × 3)
- ☐ Spread ice on the cooling box and cover it with plastic wrap. Keep the tubes chilled on ice.

**B.** **Blood sampling and subsequent processing**

④Outpatient building: Blood was collected from the subject, and dispensed into 4 blood collection tubes (1 blood count [1 ml], 1 biochemistry [5 ml], 2 EDTA [7 ml each]) and stored on ice in a cooling box. Dispose of the syringe, and bring the cooling box to the laboratory.

⑤Post-processing in laboratory at research building

1. Centrifuge 2 EDTA 7 ml bottles and 1 biochemistry 5 ml bottle for 15 minutes (1600 G, 4 °C).
2. After centrifugation, dispense plasma samples from 2 EDTA bottles into 1 microtube (3 ml) for oxytocin level quantification and 5 microtubes (1 ml). One microtube for oxytocin quantification is stored in a designated place in the freezer (−80 ° C) and later sent to Kanazawa University.
3. Store the other 5 microtubes in the freezer (−80 ° C), to be subsequently sent to the University of Tokyo. Quickly perform the processes within 30 minutes between the blood collection, and store at −80 ° C.
4. Because only blood cell components remain in EDTA tubes, cover the tubes with a recap. These are used as samples for DNA extraction.
5. Five blood collection tubes (for one blood count, one biochemistry, and three for DNA extraction) are put in the refrigerator at 4 °C together with the voucher to be picked up by LSI.

* Labeling

For blood collection tubes: Enter only the sample number on the label of the LSI voucher

Microtubes for oxytocin quantification: Enter only the sample number on the label

1 ml microtubes: Enter only the sample number on the label

For the sample number, assign the number of time-points after Viedoc ID, and further assign a or b in the case of EDTA.

For example:

The sample numbers for the EDTA tubes collected from the second blood collection at visit 6 in case 001-001 (Viedoc ID) are “001-001-2a” and “001-001-2b”.

The sample number for the biochemistry and blood count tubes collected from the first blood collection at visit 3 in case 003-012 is “003-012-1”.
