# Supplementary figures and images for: Oxytocin-induced increase in N,N-dimethylglycine and time course of changes in oxytocin efficacy for autism social core symptoms
Source: Mol Autism. 2021 Feb 23;12:15. doi: 10.1186/s13229-021-00423-z (PMC7903697; doi:10.1186/s13229-021-00423-z)

Number of detected cases

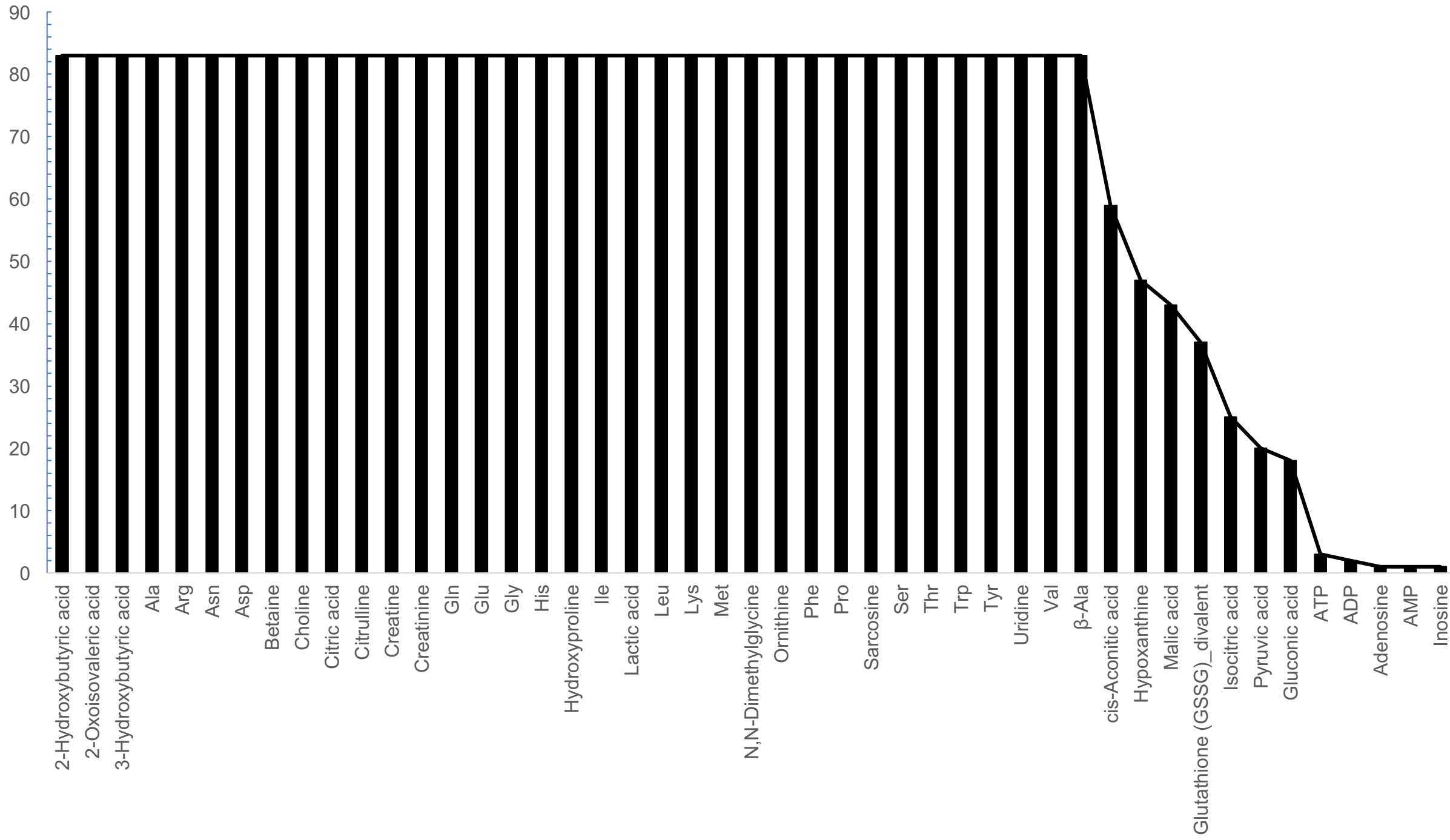

Supplement: Supplementary file 2 — Additional file 2. Supporting Table 2. Difference in changes of metabolites level between time-course change- and placebo-administered groups. [file 13229_2021_423_MOESM2_ESM.pdf]
